# Vascular pathology in multiple sclerosis: mind boosting or myth busting?

**DOI:** 10.1186/2040-7378-3-7

**Published:** 2011-07-14

**Authors:** Anne Waschbisch, Arndt Manzel, Ralf A Linker, De-Hyung Lee

**Affiliations:** 1 Department of Neurology, Schwabachanlage 6, 91054 Erlangen, Germany

## Abstract

The investigation of central nervous system vascular changes in the pathophysiology of multiple sclerosis (MS) is a time-honored concept. Yet, recent reports on changes in venous cerebrospinal outflow, the advent of new magnetic resonance imaging techniques and the investigation of immunomodulatory properties of several vascular mediators on the molecular level have added new excitement to hypotheses centering around vascular pathology as determining factor in the pathophysiology of MS. Here we critically review the concept of chronic cerebrospinal venous insufficiency in MS patients and describe new imaging techniques including perfusion weighted imaging, susceptibility weighted imaging and diffusion weighted imaging which reveal central nervous system hypoperfusion, perivascular iron deposition and diffuse structural changes in the MS brain. On a molecular basis, vascular mediators represent interesting targets connecting vascular pathology with immunomodulation. In summary, the relation of venous changes to the pathophysiology of MS may not be as simple as initially described and it certainly seems awkward to think of the complex disease MS solely as result of a simple venous outflow obstruction. Yet, the investigation of new vascular concepts as one variable in the pathophysiology of the autoimmune attack seems very worthwhile and may add to a better understanding of this devastating disorder.

## The starting point: Cerebrospinal venous insufficiency in multiple sclerosis?

Chronic cerebrospinal venous insufficiency (CCSVI) describes a vascular condition characterized by anomalies of the main extra-cranial cerebrospinal veins that interfere with normal cerebrospinal outflow [1]. These anomalies i.e. stenoses may affect the internal jugular veins, the vertebral veins or the azygous vein and can be detected by venous echo-color Doppler and invasive venography [1-4]. CCSVI has recently been reported at a high prevalence in multiple sclerosis (MS) patients and proposed as a pathogenic factor challenging the autoimmune hypothesis of the disease [1,5,6].

The idea of MS being a vascular disease is not new. In the 1930s T.J. Putnam proposed venous obstruction as the primary alteration in MS [7]. Given the venotopic localization of MS plaques, this hypothesis has been discussed on and off ever since. In 2007 an Italian group headed by P. Zamboni added new fuel to the fire by demonstrating that venous blood flow alterations can be found at a high frequency in MS patients [5]. The initial study on 89 MS patients and 60 control subjects was soon followed by a second one by the same investigators addressing venous hemodynamics in 65 patients with clinically definite MS and 235 controls [1]. Using high resolution echocolor Doppler sonography (ECD) and transcranial color Doppler sonography (TCCS). Zamboni et al. defined five parameters that-if at least two are present-allow diagnosis of CCSVI i.e. (1) reflux in the internal jugular and/or vertebral veins; (2) reflux in the deep cerebral veins; (3) B-mode evidence of internal jugular vein stenosis; (4) flow not Doppler-detectable in the internal jugular and/or vertebral veins; (5) reverted postural control of the main cerebral venous outflow pathways. He reported that the presence of venous outflow anomalies was dramatically associated with the diagnosis of MS [1]. This finding was confirmed by another study of the same group who detected CCSVI in all of 109 MS subjects but none of the 177 controls (sensitivity 100%, specificity 100%) [6]. Iron-driven inflammation was proposed as the mechanism by which CCSVI contributes to MS pathogenesis (see below). Analogous to chronic venous insufficiency of the lower legs, venous congestion is assumed to cause a higher transmural venous pressure that impairs the endothelial barrier leading to local erythrocyte extravasation. Extravascular hemolysis then may lead to iron deposition that attracts immune cells and kicks off an inflammatory cascade that culminates in MS plaque pathology [8,9].

During the last years, other groups have tried to reproduce the pioneering work of Zamboni with variable success (Table 1). Simka et al. reported the presence of CCSVI in 90% of 70 MS patients under investigation [10]. In line with that, Al-Omari and Rousan found 84% of MS patients (n = 25) to test positive for CCSVI but none of the controls (n = 25) [11]. However, other groups have failed to detect this high prevalence of CCSVI in MS questioning the concept of CCSVI as the underlying cause of the disease [12-14]. In a study by Doepp et al., none of the participants (n = 56 MS patients, n = 20 controls) fulfilled the CCSVI Doppler criteria proposed by Zamboni et al. Lack of CCSVI was also reported in a German cohort of 20 MS patients, with only one out of 20 control subjects fulfilling the CCSVI criteria [13]. Very recent data derived from a large population of MS patients does indeed suggest a higher prevalence of CCSVI in MS compared to controls, but at substantially lower numbers than those found in the original publication by Zamboni et al. 56.1% of MS patients (n = 289), 38.1% of CIS patients (n = 21) and 22.7% of healthy controls (n = 163) fulfilled the sonography criteria for CCSVI [15]. Yet, the presence of CCSVI was also high in patients with other neurological diseases (42.3%, n = 26) questioning the specificity of CCSVI in MS [14]. Of note, the prevalence of CCSVI was highest in patients with progressive MS suggesting that CCSVI may be a consequence rather than a cause of disease [15]. A possible cause-effect relationship was also challenged in a study on CIS patients: If CCSVI played a major role in MS pathogenesis, one could argue that it should be present at disease onset. However, only 16% of CIS patients tested positive for CCSVI [12]. While demographic factors like disease duration, age and sex may account for the divergent findings between the groups, methodological issues, operator-dependant differences in the interpretation of sonography findings and difficulties in operator-blinding may also play a role. Recognizing the shortfalls of venous Doppler sonography, other methods for detection of CCSVI and surrogate markers of cerebrospinal venous congestion have been evaluated. It has been hypothesized that if CCSVI was present in MS, one can assume an elevated intracranial venous pressure reflected by an increased intraocular venous occlusion pressure in MS patients [16]. Yet, a recent study on 29 MS patients and controls failed to detect such differences in venous occlusion pressure [16]. Others have argued that if MS pathophysiology was related to iron deposition in venous congestion, this should be reflected by higher ferritin levels in the cerebrospinal fluid as observed in siderosis or subarachnoidal hemorrhage. However, CSF ferritin levels were largely normal in the MS population studied [17]. Magnetic resonance venography and flow quantification was performed in a limited numbers of MS patients again demonstrating no significant differences in the extracranial venous system of MS patients and healthy controls [18-21]. Although this may argue against the vascular hypothesis of MS, one has to consider that MR may just be an inadequate method for detecting CCSVI [3,21]. Selective venous angiography is considered the gold standard among the diagnostic tests for extracranial venous stenosis (EVS). To confirm his ultrasound findings, Zamboni et al. performed invasive venography in 65 MS patients demonstrating multiple significant extracranial stenoses in all patients but in none of the 45 control subjects [1]. In contrast, the rate of EVS as shown by selective venography was significantly lower in a study by Yamout et al. [22] who pointed out several pitfalls in performing invasive extracranial venography that may lead to overestimation of venous outflow anomalies: The caliber of the internal jugular veins depends on patient's position, breathing (inspiration vs. expiration), compression by nearby structures (i.e. sternocleidomastoid muscle, esophagus, pulsating carotid arteries) and prominent valves, all of which can give a picture of pseudostenosis [22]. Accordingly, a high operator-dependency of the method can be assumed and it is not surprising that again some authors report venous stenoses in up to 97% of patients previously diagnosed with CCSVI by sonography [23], whereas others fail to do so [12]. Despite the obvious difficulties in establishing an operator-independent diagnosis of CCSVI, the vascular hypothesis of MS has recently gained a lot of attention in the media. Balloon dilatation of venous stenosis or stent implantation would in theory cure CCSVI and a number of interventional-radiologists have already started to actively promote the so-called "liberation treatment" as a therapeutic option in MS. Zamboni and colleagues have performed transluminal angioplasty in 65 patients observing a significant improvement of clinical and MRI outcome measures in an uncontrolled, unblinded trial with a mean follow-up of 18 months that lacked a sham control to assess placebo effects [3]. Safety and tolerability of endovascular treatment was recently addressed in a large trial in which balloon angioplasty was performed on 192 cases and additional stenting in another 152 cases of MS patients [23]. The procedure was reported as relatively safe with major complications (thrombotic stent occlusion, surgical removal of angioplastic balloon from the femoral vein) occurring in 1.5% of patients. Stent migration was observed in 2.3% of patients and few cases of pseudoaneurysms, gastrointestinal bleeding and atrial fibrillation were reported [23]. However there is also anecdotal evidence of stent dislocation to the right ventricle and death from cerebral hemorrhage following peri-procedural anticoagulation [24]. Up to now, there is no scientific proof of efficacy of any endovascular treatment in MS and patients should not be treated outside of randomized, controlled trials [25].

**Table 1:** Comparison of CCSVI studies

method of detection	no. of patients	no. of control subjects	main findings	CCSVI in MS	author	year	ref
TCCS	89 MS	60	alterations of intracranial venous haemodynamics in MS patients	+	Zamboni et al.	2007	[5]
TCCS-ECD (+ selective venography)	65 MS (65 MS)	235 (48)	high association of venous outflow anomalies with MS (OR43). Selective venography: multiple extracranial venous stenoses in all MS but none of the control subjects	+	Zamboni et al.	2009	[6]
TCCS-ECD	109 MS	177	presence of CCSVI (≥ 2 criteria) in all MS patients but none of the controls	+	Zamboni et al.	2009	[1]
ECD	70 MS	n/a	extracranial signs of abnormal venous outflow in 91,4% of MS patients	+	Simka et al.	2010	[10]
ECD	25 MS	25	extracranial signs of CCSVI (≥ 2 criteria) in 84% of MS patients but none of the controls	+	Al-Omari and Rousan	2010	[11]
TCCS-ECD	56 MS	20	absence of CCSVI (≥ 2 criteria) in all MS and control subjects	-	Doepp et al.	2010	[13]
TCCS-ECD	20 MS	20	absence of CCSVI (≥ 2 criteria) in all MS and 95% of controls	-	Mayer et al.	2011	[14]
TCCS-ECD	289 MS 21 CIS	189	Higher prevalence of CCSVI (≥ 2 criteria) in MS (56,1%) compared to healthy controls but low sensitivity/specificity	+/-	Zivadinov et al.	2011	[15]
TCCS-ECD (selective venography)	50 CIS (7 CIS)	170	Ultrasound criteria for CCSVI in 8 CIS patients, no venous anomalies detected by venography.	-	Baracchini et al.	2011	[12]
selective venography	31 MS 11 CIS	n/a	extracranial venous outflow anomalities infrequent in early MS, more prevalent in late MS	+/-	Yamout et al.	2010	[22]
selective venography	342 MS	n/a	venous stenoses detected in 97% of patients previously diagnosed with CCSVI by ultrasound	+	Ludyga et al.	2010	[23]

Recent studies addressing the presence of chronic cerebrospinal venous insufficiency (CCSVI) by high resolution echocolor Doppler (ECD), transcranial color Doppler sonography (TCCS) and/or selective venography.

In conclusion, the prevalence of CCSVI in MS patients was reported to range between 0% to 100% depending on the study centre and the method of detection employed. The initially proposed specificity of CCSVI detection in MS has been challenged by demonstrating its presence in healthy controls and other neurological diseases. In particular both doppler sonography and selective venography are operator-dependent and it may be difficult to ensure operator-blinding which is a major pitfall in all positive studies to date. A standardized technique, rigorous blinding and quality control of all procedures in multicenter studies are necessary to finally clarify the role of CCSVI in MS. This approach is mandatory before any attempts should be made to treat CCSVI in MS patients which at present cannot be recommended.

## The spin-off: Iron deposition and new imaging methods for vascular changes in MS

While the concept of CCSVI as prime mechanism of MS pathogenesis is increasingly challenged, the recent debate on vascular changes in MS has fostered further concepts involving vascular changes in MS pathology. Indeed, it has long been shown that demyelinated plaques in MS are centred around small venules [26] and that inflammatory cell infiltrates are localised around small or medium sized CNS veins [27]. Recently, perivascular iron deposition has been implicated in the pathogenesis of MS. This mechanism has also been proposed as a molecular consequence of CCSVI leading to inflammation and plaque generation although to date, this concept remains largely unproven (see above).

In the central nervous system (CNS), iron is not only found around vessels, but is also localized to oligodendrocytes. Iron ions play an important role for vital cell processes and contribute to oxygen consumption, ATP production, DNA synthesis and xenobiotic metabolism [28]. Thus, iron metabolism is controlled by a large number of enzymes and proteins. A malfunction in this sensitive system may result in an excess of free ionic Fe^2+ ^which is potentially detrimental to the surrounding environment and finally may lead to the generation of harmful free radicals [29]. In the past, the functional role of iron for myelination and demyelination has been investigated in different animal models. Importantly, iron mediated oxidative stress is likely involved in the pathogenesis of demyelination [30], and venous haemorrhage with iron deposition has been reported in several demyelinating diseases other than MS as for example Hurst acute haemorrhagic leukencephalitis [31]. Yet to date, there are only few data on iron deposition in experimental autoimmune encephalomyelitis (EAE), the model disease mimicking many aspects of MS. Iron deposition and related infiltration of macrophages and granulocytes were observed in SJL mice suffering from myelin basic protein induced EAE. The source of such iron deposits might be explained by the breakdown of the blood brain barrier permitting iron containing plasma proteins and red blood cells to penetrate into the brain [32]. However, on a histological level, hemorrhagic lesions are only found in very severe EAE, e.g. in myelin oligodendrocyte glycoprotein induced EAE of congenic 1AV1 rats.

In first studies on specimen from MS patients in 1982, Craelius and colleagues found iron deposits close to demyelinating plaques in five of five MS cases, but not in any controls [33]. In contrast, a subsequent post-mortem study investigating 13 MS patients could not confirm this observation [34]. Adams and colleagues analysed brain tissue of 70 MS patients and observed that veins localised in MS plaques were more extensively damaged than expected [27]. These alterations were seen as deposits of fibrin and fibrinoid in the vessel wall, frequent hemorrhages or residual hemosiderin as evidence of past haemorrhage as well as occlusion of plaque veins due to thrombosis, which correlated with iron deposition in only 21 patients. Poor detection of iron deposits in the tissue might be explained by the staining method used. This procedure was optimized with the help of diaminobenzamide technique [35] and this modified protocol may enable to prove the existence of iron in a finer matrix e.g. in oligodendrocytes [36-38].

In 1987, iron deposition in MS brains was suggested as reduced signal intensity on T2 weighted magnetic resonance (MR) images of the basal ganglia [39]. Nearly a decade later, studies on brain autopsies from 46 patients with definite MS revealed a significant correlation between areas of prominent low intensity in T2 weighted imaging and sites of iron deposition [39]. These results were repeatedly confirmed with newer MR techniques. Later, the extent of gray matter T2 hypo-intensity was correlated with EDSS progression as indicator of disability [40]. Well in line with this concept, gray matter T2 hypo-intensity and iron deposition also correlated with the extent of brain atrophy in MS patients [41].

Nowadays, iron deposition may easily be detected *in vivo *using susceptibility-weighed imaging (SWI). This new technique (including a three dimensional, long echo time, gradient-echo sequence) was developed in the late 1990s and was initially employed to assess and to depict small veins in the CNS [42-44]. Although this method was already well established in clinical radiology for more than 10 years, first investigations of MS patients were not performed until nearly a decade later. Recently, a controversially discussed study by Zivadinov and Zamboni correlated venous hemodynamic parameters and iron deposition. This pilot case-controlled trial investigated 16 patients with a confirmed relapsing remitting MS (RRMS). Each patient fulfilled the criteria of CCSVI and iron deposition was significantly related to venous obstruction as assessed by the "Zamboni criteria" [1,21]. While this relation of iron deposition to CCSVI awaits further systematic investigation by independent groups, iron deposition may to some degree correlate with disability and atrophy in MS patients. Here, SWI may enable a better characterization of lesions demonstrated by conventional MR imaging.

Today, conventional magnetic resonance imaging (MRI) belongs to the routine diagnostic work-up of MS patients and innovative MRI techniques have contributed to the recognition of diffuse brain damage in MS patients. Besides classical protocols depicting white matter alterations, advanced MRI techniques such as proton MR spectroscopy (MRS), magnetization transfer imaging (MTR) and diffusion tensor imaging have added valuable new information on tissue changes in MS brains. In particular, proton MRS and MTR have supported the concept of a diffuse pathology involving the normal appearing white matter (NAWM) and grey matter (GM) in MS pathology. Moreover, perfusion weighted imaging (PWI) and diffusion weighted imaging (DWI) have recently contributed to a better understanding of vascular changes in MS pathophysiology. The DWI technique employs the variability of "Brownian motion" of water molecules in brain tissue. In the brain parenchyma, their rate of movement (or "diffusion") is restricted due to tissue structures such as axons or oligodendrocytes, respectively. Thus DWI enables the characterisation of distinct pathological substrates and DWI allows the detection of MS pathology in NAWM as well as grey matter. In MS patients, diffusivity of normal appearing grey matter is increased, and changes in diffusion correlate with cognitive deficits in MS patients. Moreover, diffusion changes might be a sensitive marker for disease progression and may thus supplement present MRI standards [45].

Parameters of interest in perfusion weighted imaging (PWI) include vascular mean transit time, cerebral blood volume (CBV) as well as cerebral blood flow (CBF). Dynamic susceptibility contrast-enhanced MRI is based on the acquisition of images during a bolus of contrast agent through the vessels of the brain. In the late 1990s, the hypothesis arose that leukocyte trafficking into the parenchyma may be initiated through a reduction of blood flow [46]. Indeed, PWI analyses detected a significantly decreased cerebral blood flow of roughly 50% and a more than twofold prolonged mean transit time throughout the NAWM in RRMS patients [47]. Moreover, CBF and CBV were also reduced in patients with primary progressive MS [48]. The same phenomenon was observed in the grey matter of MS patients which might be caused by the same mechanism underlying the reduced CBF in the NAWM [49]. Recently, these observations were confirmed by an independent group [50]. In summary, hypoperfusion has been reported in lesions, NAWM and GM of MS patients. These observations were correlated with the disease progression of MS patients. These data imply a possible contribution of vascular changes to the generation of MS lesions in CNS white matter: Ge and co-workers suggested that hypoperfusion in the NAWM may be caused by a vasculopathy in the context of perivascular inflammation that occurs in focal MS lesions [49]. Yet on a microscopic level, thrombosis of microvessels is only detected in exceptional cases [51]. To date, it remains to be elucidated if CNS hypoperfusion in MS is really causally related to, or rather a consequence of disease pathogenesis.

## The molecular basis: Vascular mediators in autoimmune demyelination

**Figure 1:**
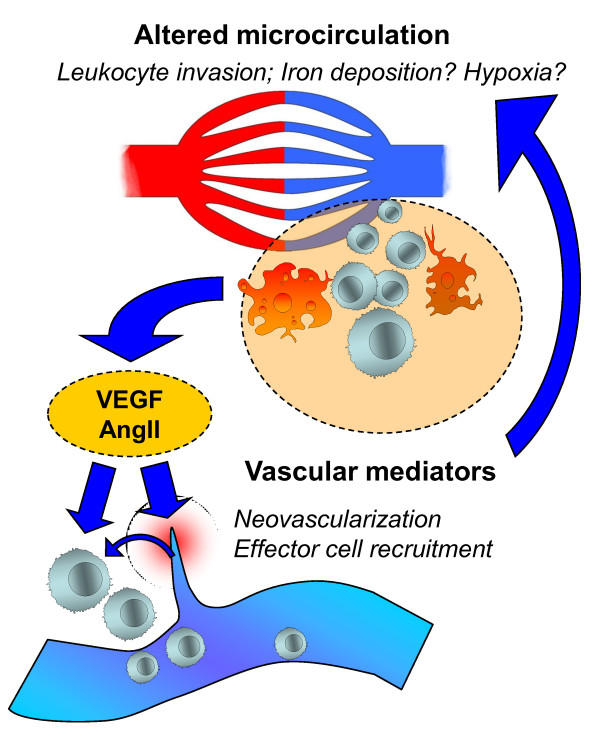
**Scheme depicting the possible interplay between vascular mediators and changes in microcirculation during autoimmune inflammation of the CNS**. Altered microcirculation may lead to iron deposition and increased leukocyte infiltration. In turn, pleiotropic factors such as vascular mediators released by immune cells and the activated endothelium can cause neovascularization and lead to the recruitment of further effector cells. This mechanism may be part of a feed-forward loop that perpetuates the inflammatory process in MS.

While several studies on the role of vascular changes in MS focused on imaging techniques including Doppler and MRI, further approaches concentrated on the role of vascular mediators in autoimmune demyelination. Hormones, adhesion molecules and enzymes primarily involved in processes of vascular physiology such as haemostasis, blood pressure control, angiogenesis and endothelial functioning are often also key players in vascular-immune interactions and immunomodulation. This section outlines the relevance of two prototypic vascular regulatory systems in autoimmune inflammation of the CNS (Figure 1), describes their potential beneficial and harmful characteristics and discusses their potential as new therapeutic targets.

### Vascular Endothelial Growth Factor Signalling

Vascular endothelial growth factor (VEGF) is a heparin-binding, pro-angiogenic glycoprotein that in humans exists in four secreted isoforms (VEGF A-D). VEGF-A and VEGF-B exclusively promote vascular neogenesis via their tyrosinkinase-receptors VEGFR1/Flt-1 and VEGFR2/KDR, while VEGF-C and VEGF-D additionally promote lymphangiogenesis via VEGFR4/Flt-4. Herein, VEGF refers to VEGF-A.

There is a feed-forward interdependence of angiogenesis and chronic inflammation. Inflammatory mediators induce angiogenesis. In turn, new blood vessels facilitate immune cell migration to the site of inflammation and increase the capacity for immune-cell adhesion, cytokine and chemokine production [52]. The regulation of VEGF and its biological actions replicate this co-dependence on the molecular level. VEGF expression is induced by hypoxia, a frequent condition in inflammatory lesions [53]. The proinflammatory cytokines interleukin-1ß, tumor necrosis factor-alpha and interleukin-18 [54,55] directly promote VEGF production in various cells types. VEGF secreted by vascular endothelial cells is chemotactic for T-cells and macrophages [56]. Vice-versa, macrophages themselves are producers of VEGF-C and thereby actively influence vascular angiogenesis and lymphangiogenesis [57]. The intracellular response to VEGF involves the transcription factor "nuclear factor of activated T-cells" (NF-AT) [58]. Thereby, compared to other angiogenic factors like basic fibroblast growth factor (bFGF) and epidermal growth factor (EGF), VEGF activates a broader transcriptional response in target cells and induces numerous inflammation-related genes, such as the chemokine ligands CXCL1, CXCL2, interleukin-8 and tissue-factor [58,59].

Not surprisingly, alterations in the VEGF/VEGFR system are observed in various inflammatory autoimmune diseases such as lupus erythematosus, inflammatory bowel disease, psoriasis, rheumatoid arthritis and MS (reviewed in [60]). In MS, VEGF serum levels are elevated during relapses [61] and its receptor VEGFR2 (KDR/Flk-1) is highly expressed in active MS lesions, paralleled by an increased number of microvessels [62]. Breakdown of the blood-brain barrier is a significant event in MS lesions and the capability of VEGF to downregulate claudin-5 and occludins, key components of tight junctions, promotes BBB breakdown in murine MOG-EAE [54]. Additional EAE studies confirm the presence of increased angiogenesis and proinflammatory VEGF signalling in autoimmune CNS inflammation [62-66]. Conversely, one study also reports the downregulation of VEGF in astroglia of Lewis rats suffering from EAE [67].

Anti-VEGF therapy has originally evolved as a treatment against solid tumor growth and bevacizumab, a human monoclonal antibody to VEGF has been approved for the treatment of various types of cancer, as well as angiogenic ocular diseases [68]. The proinflammatory properties of VEGF render it a potential therapeutic target in autoimmune disease. Although human clinical studies on autoimmune diseases have not yet been reported, antagonizing VEGF signalling was effective in animal models of psoriasis [69,70], rheumatoid arthritis and [71] MS [64]. Notably, antagonizing VEGFR-2 ameliorates murine MOG-EAE only in the acute, but not in the chronic phase [64] which is well in line with a predominant anti-inflammatory mode of action of this treatment. Therefore, targeting the VEGF pathway in MS seems a promising approach to treat the early, inflammation-driven phase of MS (RRMS) rather than the chronic-progressive phase of the disease (SPMS).

### Renin-Angiotensin-System

The renin-angiotensin-system (RAS) is a master regulator of blood pressure, fluid balance and ion homeostasis. The main RAS pathway consists of a proteolytic cascade that generates the peptide hormone angiotensin II (AngII) from its precursors angiotensinogen and angiotensin I through sequential cleavage by renin and angiotensin-converting-enzyme (ACE). Two side-pathways of the RAS have more recently been discovered with their messengers being the AngII metabolites AngIV and Ang1-7. While the biological actions of the alternative angiotensin peptides are still debated, the main RAS hormone AngII is well studied with regard to its role as a haemodynamic regulator. This effect is mostly mediated by binding to metabotropic angiotensin II type 1 receptors (AT1). AT1 signalling in turn facilitates a multitude of synergistic pressor effects like contraction of vascular smooth muscle cells, renal sodium reabsorption and thirst arousal.

Through its highly pleiotropic actions, AngII also favours inflammatory events. It increases vascular permeability via the release of prostaglandins [72,73] and VEGF [74], whereas VEGF is itself a highly pleiotropic, inflammatory mediator (see above). AngII contributes to the recruitment of inflammatory cells into the tissue through the regulation of endothelial adhesion molecules [75,76] and chemokines in various cell types [77-79]. Moreover, leukocytes express a complete set of RAS components and are capable of delivering AngII to inflammatory sites [78,80,81] where it may perpetuate inflammation.

Enhanced RAS activity and AngII levels are major culprits in hypertensive disease and hypertensive end-organ damage [82,83] Interestingly, experimental *in vivo *studies suggest that T-cells are necessary to transform elevated AngII levels into vascular pathology [82], providing a link to T-cell mediated autoimmune disease.

Indeed, alterations in the RAS have been detected in MS patients including decreased CSF AngII levels [84] as well increased serum ACE levels [85]. RAS components, including AT1 are specifically up-regulated in MS brain lesions [86]. Complementing the findings in MS, the mechanistic role of the RAS in CNS autoimmune disease was explored in murine EAE. Antigen-specific CD4+ T-cells strongly up-regulate AT1 following induction of disease with a proteolipid lipoprotein peptide (PLP 139-151). Inhibition of ACE by lisinopril, an approved drug for the treatment of arterial hypertension, led to a shift in PLP-responsive T-cell populations, favouring expansion of regulatory T-cells over auto-aggressive Th1/Th17 cells. Clinical symptoms of EAE were reduced in lisinopril treated animals. This effect was even more pronounced when T cells from either lisinopril or vehicle-treated animals were transferred to recipient mice prior to EAE induction [86], highlighting a dominant role of the T cell as AngII-target in EAE. In a similar murine EAE model (MOG-EAE), macrophages upregulate AT1 transcripts up to 1000-fold. Blockade of AT1 by losartan impaired macrophage mobility *in-vitro *and greatly reduced splenic macrophage cell counts as well as the number of CNS-invading macrophages *in vivo*. Accordingly, clinical symptoms of MOG-EAE were reduced in losartan-treated animals. Notably, the modulation of EAE was independent of blood pressure effects caused by the treatment [87].

In summary, elevated RAS activity is observed in MS and anti-AngII therapy ameliorates clinical symptoms of EAE. Direct modulation of macrophages and T-cells contributes to the beneficial effects of anti-AngII therapy in EAE. Additional contributing factors which may include the AngII metabolites AngIV and Ang1-7 currently await further exploration. Finally, the application of long-approved anti-hypertensive drugs with a well known safety profile as new therapeutic option in MS is an appealing prospect.

## Conclusion

While the concept of CCSVI has gained much attention in the field of MS research and in particular among MS patients, there is increasing evidence that the relation of venous changes to the pathophysiology of MS may not be as simple as initially described. Most importantly, new MR imaging techniques add to the notion of vascular changes in MS, yet again raise doubts whether these alterations are cause or rather consequence of the disease process. On a more refined level, iron deposition as well as vascular mediators add to our molecular understanding of the complex network of autoimmune processes which undoubtedly underlie the formation on a demyelinating plaque in the CNS.

## Competing interests

The authors declare that they have no competing interests.

## Authors' contributions

AW has been involved in literature search and writing as well as drafting the manuscript. AM has been involved in in literature search and writing as well as drafting the manuscript. RL has been involved in drafting the manuscript and has given final approval. DL has been involved in in literature search and writing as well as drafting the manuscript and has given final approval. All authors read and approved the final manuscript.
